# Deformation Effect on SUV_max_ Changes in Thoracic Tumors Using 4-D PET/CT Scan

**DOI:** 10.1371/journal.pone.0058886

**Published:** 2013-03-14

**Authors:** Tzung-Chi Huang, Yao-Ching Wang

**Affiliations:** 1 Department of Biomedical Imaging and Radiological Science, China Medical University, Taichung, Taiwan; 2 Division of Radiation Oncology, China Medical University Hospital, Taichung, Taiwan; The University of Chicago, United States of America

## Abstract

Respiratory motion blurs the standardized uptake value (SUV) and leads to a further signal reduction and changes in the SUV maxima. 4D PET can provide accurate tumor localization as a function of the respiratory phase in PET/CT imaging. We investigated thoracic tumor motion by respiratory 4D CT and assessed its deformation effect on the SUV changes in 4D PET imaging using clinical patient data. Twelve radiation oncology patients with thoracic cancer, including five lung cancer patients and seven esophageal cancer patients, were recruited to the present study. The 4D CT and PET image sets were acquired and reconstructed for 10 respiratory phases across the whole respiratory cycle. The optical flow method was applied to the 4D CT data to calculate the maximum displacements of the tumor motion in respiration. Our results show that increased tumor motion has a significant degree of association with the SUV_max_ loss for lung cancer. The results also show that the SUV_max_ loss has a higher correlation with tumors located at lower lobe of lung or at lower regions of esophagus.

## Introduction

PET/CT, which provides both functional and anatomical images simultaneously, has demonstrated to be advantageous for the diagnosis and treatment of cancer patients [1]–[4]. PET can be used in patient diagnosis for the initial tumor staging and restaging. With respect to the use of radiotherapy in treatment planning, the incorporation of PET information aids in delineation of the target volume, allowing for adequate dose coverage and minimal toxicity to normal tissues; PET also facilitates monitoring of the therapy response. However, the long acquisition time of PET imaging compared to CT anatomic imaging may cause problems in data registration for a diagnostic PET/CT scan. For lung cancers, additional challenges due to respiration in the thoracic section complicate the interpretation of PET images and also lead to blurring and mis-registration artifacts between PET and CT scans [5]. The blurring of PET from the motion of respiration results in two main artifacts, an increase in the tumor size and reduction of standardized uptake value (SUV).

A PET image is considered to be the time-averaged map of the SUV because the acquisition covers several breathing cycles. Respiratory motion can blur the SUV and lead to additional signal reduction. As a result, changes in the SUV maxima are caused by the respiratory pattern rather than by the original tumor fluorodeoxyglucose (FDG) uptake. Gated lung (4D) PET scanning was developed to reduce the effects of blurring in the detection of FDG uptake [6]–[9]. Combined with 4D CT acquisition, 4D PET can provideaccurate tumor location information as a function of the respiratory phase in PET/CT imaging [8]. Several phantom studies have investigated the effects of motion on PET scans [5], [9]–[10]. 4D PET (gated) whole body scans that avoid signal loss and minimize the effects of tumor motion were reported in previous studies [5]. Callahan et al. demonstrated significant association between lesion displacements and decreases in the SUV [9]. However, previous studies only investigated the effect of motion on the SUV changes in phantom. They did not directly explore the effect of the tumor motion inpatients and did not consider three-dimensional motion and deformation in the anterior-posterior, lateral and superior-inferior directions.

In this study, we investigated thoracic tumor motion with respiratory gated (4D) CT imaging and assessed the effect of thoracic tumor motion on the SUVs in 4D PET imaging using a clinical patient cohort. The SUV difference between 3D and 4D PET imaging due to motion of respiration was also evaluated.

## Materials and Methods

### Study Subjects

Under a protocol approved by the IRB at the China Medical University Hospital, Taiwan (DMR98-IRB-171-2), 4D FDG-PET/CT data were obtained from twelve radiation oncology patients with thoracic cancer including five lung cancer patients and seven esophageal cancer patients prior to radiation therapy. The clinical characteristics of these twelve patients are presented in Table 1. All patients signed written, informed consent. The 4D PET scans were performed during the same session, immediately after the 3D PET scans, which were acquired for a clinical purpose, using a single FDG dose. The 4D CT scans were used to contour the internal target volumes (ITV) for treatment planning, while the 4D PET scans were analyzed retrospectively only for the purpose of research.

**Table 1:** Clinical characteristics of the study subjects and tumors.

Patient #	Sex	Age(y)	Histology	Location	Type	Stage
1	F	57	Adenocarcinoma	Lung Cancer/ Left upper lobe	Lymph node	II
2	M	59	Squamous cell carcinoma	Lung Cancer/ Left lower lobe	Lymph node	III
3	M	63	Squamous cell carcinoma	Lung Cancer/ Right lower lobe	Lymph node	III
4	M	61	Squamous cell carcinoma	Lung Cancer/ Right lower lobe	Lymph node	III
5	M	64	Adenocarcinoma	Lung Cancer/ Right lower lobe	Lymph node	III
6	M	42	Squamous cell carcinoma	Esophageal cancer (Upper)	Lymph node	II
7	M	37	Dysplastic squamous cells	Esophageal cancer (Middle to lower)	Lymph node	III
8	M	40	Squamous cell carcinoma	Esophageal cancer (Middle)	Lymph node	III
9	M	58	Squamous cell carcinoma	Esophageal cancer (Lower)	Lymph node	III
10	M	53	Squamous cell carcinoma	Esophageal cancer (Lower)	Lymph node	IV
11	M	53	Squamous cell carcinoma	Esophageal cancer (Lower)	Primary	II
12	M	44	Squamous cell carcinoma	Esophageal cancer (Upper )	Primary	II

### 4D PET/CT scan protocol

The data were acquired using GE PET/CT-16 slice, Discovery STE (GE Medical System, Milwaukee, Wisconsin USA) incorporated with a Varian real-time position management (RPM system, Varian Medical Systems, Inc. Palo Alto, CA) for respiratory motion tracking. The 4D sinograms were sorted using the amplitude mode and then reconstructed for each individual phase, and the 4D scans were reconstructed on a 512×512 image matrix. The pixel size in the transaxial slice of the 4D CT images was approximately 0.98×0.98 mm^2^, and the slice thickness was 2.5 mm. The 4D CT image sets were acquired and reconstructed for 10 respiratory phases across the entire respiratory cycle. The 10 respiratory phases were labeled as T5%, T15%, … T95% phases, with the T5% phase approximately corresponding to the normal end-inspiration and the T55% to the end expiration.

FDG with an activity of around 10 mCi was administered to the patients, and the PET/CT scan session was performed approximately 40 minutes after injection. Patients were placed with their arms up, which matches the treatment simulation position. A thoracic 3D PET scan of two bed positions was performed initially with an 8- to 10-minute acquisition per bed position. The 3D scans were reconstructed on a 128×128 image matrix with a voxel size of 4.46×5.46×3.27 mm^3^. A respiratory gated 4D PET scan of the whole lung was performed with the patient in the same position using the RPM respiratory gating system immediately after the 3D PET scan. A 3D CT scan was used for attenuation correction for both the 3D and 4D PET scans. The ordered subset expectation maximization reconstruction method was used with 2 iterations and 20 subsets for the 3D and the 4D scans. A post-reconstruction Gaussian filter was applied with a full-width at half-maximum of 6 mm for the 3D scan and 7 mm for the 4D scan.

### Tumor motion estimation

Deformable image registration is required to generate voxel-to-voxel deformation matrices among the involved CT image sets [11]. We developed a four-dimensional deformable image registration algorithm based on the optical flow method (OFM), which links all expiratory phases in the 4D CT. The validation and several successful applications were reported in previous studies [12]–[14]. The OFM calculation equation is



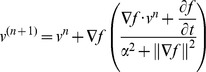



where *n* is the number of iterations (100 in all estimations), *v(n)* is the average velocity derived from the surrounding voxels, *f* is the image intensity, and *α* is the weighting factor that empirically set at 5 for the CT imaging modality. In this study, *v* was calculated for each voxel of the contoured tumor in the anterior-posterior (AP), lateral (LAT) and superior-inferior (SI) directions. The average tumor deformation (TD) for each voxel is defined as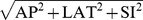. An optical flow program was utilized to calculate the maximum displacements of the TD during the respiratory phases in a 4-D CT data set. The maximum tumor motion during the entire respiratory cycle was acquired between the end-inhalation phase (T5%) and the end-exhalation phase (T55%).

### Data analysis

A region of interest (ROI) was delineated for each tumor individually on the 3D PET scan, at each of the ten phases of the 4D PET scan and on the 4D CT scan. The threshold level was set at 40% of the maximum SUV (SUV_max_) to segment the tumor [8], [15]–[17].

We measured the SUV maximum within the ROI for the 3D scan, SUV_3D max_, and the highest intensity in the ten phases of the 4D scan, SUV_4D max_. To investigate the effect of tumor motion on the results, we also defined a quantity to indicate the percentage difference (PD) in the SUV_max_ between the 3D and 4D scans.



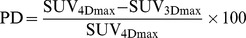



Finally, the Pearson correlation coefficient between the deformation and PD was calculated using MedCalc software (Med Calc Software, Mariakerke, Belgium).

## Results

**Figure 1:**
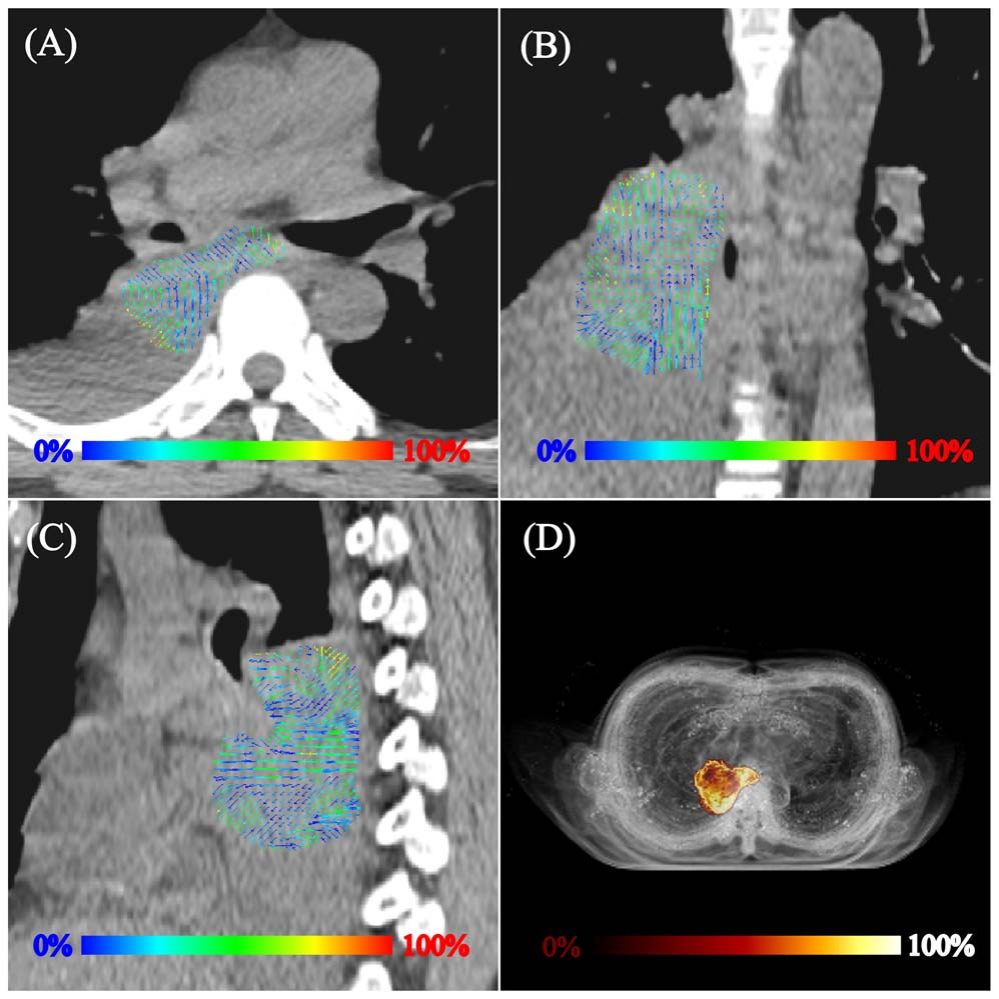
The projection of the tumor deformation vectors with the color scale superimposed on the CT scan for lung cancer patient 3 in the (A) transverse, (B) coronal and (C) sagittal views. (D) A rendering of the tumor deformation with a scout view of the thoracic CT for the same patient.

**Figure 2:**
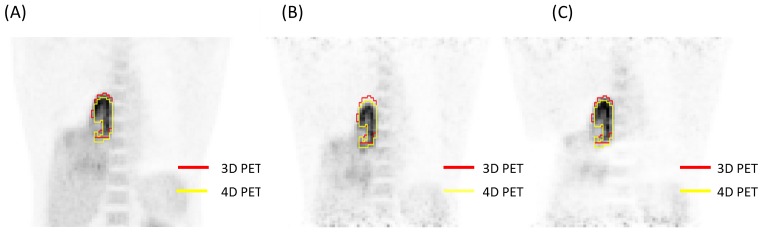
One example selected by the maximum PD is shown in the coronal view of the PET scan. (A) Differences observed between the tumor depiction in the 3D and 4D PET scans. The 4D contour was consistently lager in the SI direction. FDG-avid regions in the (B) T5% and (C) T55% respiratory phases moving outside the boundary depicted by the 3D contour.

**Figure 3:**
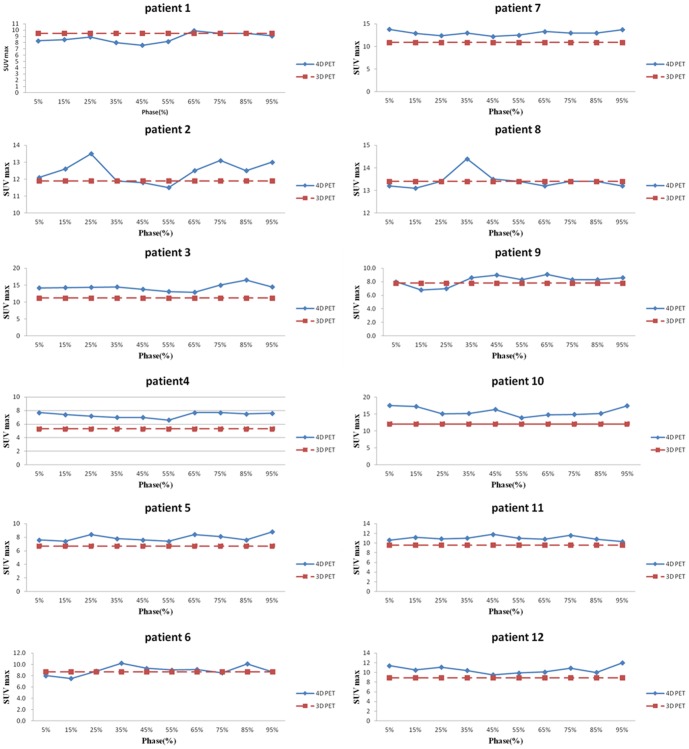
The comparison of the SUV_max_ with every phase in the 4D and 3D PET scans for each patient.

**Figure 4:**
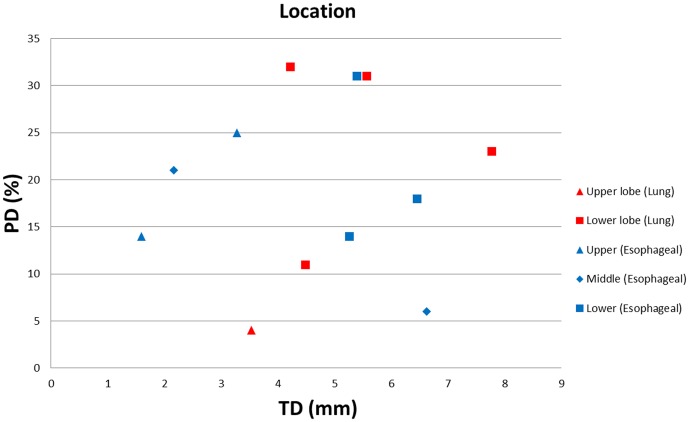
The SUV_max_ percentage difference (PD%) between the 3D and 4D PET scans, comparing the tumor deformation (TD) with the location of tumors, including lung and esophageal cancer.

Figure 1 shows the projection of the tumor deformation vectors with the color scale superimposed on the CT for lung cancer patient 3 in the (A) transverse, (B) coronal and (C) sagittal views. (D) shows a rendering of the TD with a scout view of the thoracic CT for the same patient. Figure 2(A) illustrates the differences observed between the tumor depiction on 3D and 4D PET scans. One example selected by maximum PD is shown in the coronal view of the PET scan. The 4D contour was consistently larger in the SI direction. FDG-avid regions in the T5% and T55% respiratory phases moving outside the boundary depicted using 3D contours were observed in Figures 2(B) and 2(C). Figure 3 demonstrates the comparison of the SUVmax with every phase in the 4D and 3D PET scans for each patient. Table 2 shows the SUV_3D max_, SUV_4D max_ and PD for each tumor from the 3D and 4D PET scans, along with the deformation of the tumor (T5% to T55%) by the OFM calculation. The range of deformation for the TD among the tumors varies from 1.00 to 7.77 mm. The maximum values for lung and esophageal cancers were 7.77 and 6.46 mm, respectively. The maximum SUV was 13.4 for the 3D PET scan and 17.5 for the 4D scan. The maximum percentage difference between the 3D and 4D PET scans (PD) was 32, which was found in patient 3, who had lung cancer. The correlations between the PD and TD were 0.4 for the lung cancer group (patients #1–5) and −0.2 for esophageal cancer patients (patients #6–12). For the individual direction of the tumor deformation, the correlations were 0.59, 0.48, and 0.31 for lung cancers and −0.43, −0.7, and 0.14 for esophageal cancers in the AP, LAT, and SI directions, respectively. In Figure 4, patients with lower lung lobe tumors have lager SUV_max_ differences between the 4D PET and 3D PET scans.

**Table 2:** The characteristics of each tumor used in the analysis.

Patient #	Deformation of tumor motion(mm)				SUV_3DMAX_	SUV_4DMAX_	PD (%)
	AP	LAT	SI	TD			
1	0.56±0.26	0.66±0.37	3.39±1.04	3.54±0.91	9.5	9.9	4
2	0.88±0.77	0.87±0.85	4.05±2.13	4.49±2.29	11.9	13.5	11
3	0.77±0.64	0.65±0.44	3.99±1.80	4.22±1.95	11.2	16.5	32
4	1.87±1.36	1.67±1.13	4.55±2.67	5.57±3.03	5.3	7.7	31
5	0.94±0.80	0.94±0.80	7.55±2.46	7.77±2.29	6.7	8.8	23
6	0.49±0.33	0.84±0.59	1.00±0.75	1.60±0.82	8.7	10.2	14
7	0.97±0.55	0.60±0.46	1.69±0.66	2.17±0.68	10.9	13.8	21
8	1.73±1.24	3.23±1.07	5.14±2.53	6.63±2.66	13.4	14.4	6
9	1.64±0.97	2.28±1.26	4.04±2.19	5.26±2.37	7.8	9.1	14
10	1.19±0.87	1.22±0.75	4.94±2.11	5.40±2.20	12	17.5	31
11	0.78±0.62	1.02±0.58	6.27±2.14	6.46±2.09	9.6	11.8	18
12	0.44±0.17	0.28±0.21	3.21±1.27	3.28±1.34	8.9	12	25

## Discussion

The SUV_max_ of moving tumors as measured with 4D PET scanning is usually higher than for the 3D scan as a result of averaging a long acquisition time. There are reports that signal loss in 3D PET scans is caused by the amount of displacement and pattern of respiration motion, and the 4D PET scan is able to recover most of the loss induced by respiratory motion [5], [9], [12]. We observed a similar phenomenon, which is shown in Figure 3. However, 4D PET has a lower SUVmax in one case due to the statistical uncertainty.

It is fairly obvious that tumors closer to the diaphragm move with a larger amplitude in the SI direction for lung cancer; therefore, we expect that there is a correlation between the magnitude of deformation and the tumor location. In Table 2, our results show that the deformation in the SI direction is larger than in the AP and LAT directions, which contributes the most to tumor motion. This finding is consistent with previous studies [12], [18]. In our statistical analysis, the correlation coefficient of 0.4 between the PD and TD in lung cancer may affect the degree to which the 4D PET results differ from 3D PET.

Figure 4 demonstrates that patients with lower lung lobe tumors have larger SUV_max_ differences between the 4D PET to 3D PET scans. Therefore, the tumor location correlates with the deformation of the tumor and, thus, with the SUV_max_ changes. These observations are in accordance with the studies by Aristophanous and Callahan *et al.*, which had larger patient populations and also contained phantom experiments [8]–[9]. For example, a significant loss in the SUV_max_ was observed in the tumors located in the lower lobe in their study. However, the SUV_max_ changes of tumor in esophagus were not correlated with either the TD and PD or PD and tumor location. This information can be used in clinical practice to decide which patients have the potential to benefit from 4D PET. If substantial tumor motion is revealed in 4D CT, a 4D PET scan should be prescribed instead of a 3D PET scan to more accurately distinguish an FDG-avid tumor from an adjacent non-avid tissue or fluid.

## Conclusions

In this study, we have attempted to show the SUV_max_ difference between 3D and 4D PET scans with deformation and with tumor localization in thoracic cancer patients. Our results show that increased tumor deformation has a significant association with the SUV_max_ loss for lung cancer. The results also show that tumor location in the lower lung lobe is correlated with a decrease in the SUV_max_, while tumors in the esophagus are not correlated with the SUV_max_, the tumor deformation or tumor location in this limited clinical patient cohort.

## References

[1] Wahl RL, Jacene H, Kasamon Y, Lodge MA (2009) From RECIST to PERCIST: evolving considerations for PET response criteria in solid tumors. J Nucl Med. 50: 122S–50S. 10.2967/jnumed.108.057307 PMC2755245 19403881

[2] MacManus M, Nestle U (2009) Rosenzweig KE, Carrio I, Messa C, et al. Use of PET and PET/CT for radiation therapy planning: IAEA expert report 2006–2007. Radiother Oncol. 91: 85–94. 10.1016/j.radonc.2008.11.008 19100641

[3] Lardinois D, Weder W, Hany TF, Kamel EM, Korom S, et al (2003) Staging of non-small-cell lung cancer with integrated positron-emission tomography and computed tomography. N Engl J Med. 348: 2500–2507. 10.1056/NEJMoa022136 12815135

[4] Abramyuk A, Tokalov S, Zöphel K, Koch A, Szluha Lazanyi K, et al (2009) Is pre-therapeutical FDG-PET/CT capable to detect high risk tumor subvolumes responsible for local failure in non-small cell lung cancer? Radiother Oncol. 91: 399–404. 10.1016/j.radonc.2009.01.003 19168248

[5] Park SJ, Ionascu D, Killoran J, Mamede M, Gerbaudo VH, et al (2008) Evaluation of the combined effects of target size, respiratory motion and background activity on 3D and 4D PET/CT images. Phys Med Biol. 53: 3661–79. 10.1088/0031-9155/53/13/018 18562782

[6] Nehmeh SA, Erdi YE, Pan T, Pevsner A, Rosenzweig KE, et al (2004) Four-dimensional (4D) PET/CT imaging of the thorax. Med Phys. 31: 3179–3186. 10.1118/1.1809778 15651600

[7] Wolthaus JW, Van Herk M, Muller SH, Belderbos JS, Lebesque JV, et al (2005) Fusion of respiration-correlated PET and CT scans: Correlated lung tumour motion in anatomical and functional scans. Phys Med Biol. 50: 1569–1583. 10.1088/0031-9155/50/7/017 15798344

[8] Aristophanous M, Berbeco RI, Killoran JH, Yap JT, Sher DJ, et al (2012) Clinical utility of 4D FDG-PET/CT scans in radiation treatment planning. Int. J. Radiation Oncology Biol. Phys. 82: e99–e105. 10.1016/j.ijrobp.2010.12.060 21377285

[9] Callahan J, Binns D, Dunn L, Kron T (2011) Motion effects on SUV and lesion volume in 3D and 4D PET scanning. Australas Phys Eng Sci Med. 34: 489–495. 10.1007/s13246-011-0109-x 22081269

[10] Pevsner A, Nehmeh SA, Humm JL, Mageras GS, Erdi YE (2005) Effect of motion on tracer activity determination in CT attenuation corrected PET images: a lung phantom study. Med Phys. 32: 2358–62. 10.1118/1.1943809 28493572

[11] Horn BKP, Schunck BG (1981) Determining optical flow. Artif Intell. 17: 185–203.

[12] Guerrero T, Zhang G, Huang TC, Lin KP (2004) Intrathoracic tumour motion estimation from CT imaging using the 3D optical flow method, Phys Med Biol. 49: 4147–4161. 10.1088/0031-9155/49/17/022 15470929

[13] Huang TC, Greta S.P Mo, Wang SJ, Wu TH, Zhang G (2011) Attenuation correction of PET images with interpolated average CT for thoracic tumors. Phys Med Biol. 56: 2559–2567. 10.1088/0031-9155/56/8/014 21444973

[14] Huang TC, Liang JA, Dilling T, Wu TH, Zhang G (2010) Four-dimensional dosimetry validation and study in lung radiotherapy using deformable image registration and Monte Carlo techniques, Radiation Oncology. 5: 45. 10.1186/1748-717X-5-45 PMC2890615 20509955

[15] Cerfolio RJ, Bryant AS, Winokur TS, Ohja B, Bartolucci AA (2004) Repeat FDG-PET after neoadjuvant therapy is a predictor of pathologic response in patients with non-small cell lung cancer. Ann Thorac Surg. 78: 1903–9. 10.1016/j.athoracsur.2004.06.102 15560998

[16] Aristophanous M, Berbeco RI, Killoran JH, Yap JT, Sher DJ, et al (2012) Clinical utility of 4D FDG-PET/CT scans in radiation treatment planning. Int. J. Radiat.Oncol. Biol. Phys. 82: 99–105. 10.1016/j.ijrobp.2010.12.060 21377285

[17] Bradley J, Thorstad WL, Mutic S, Miller TR, Dehdashti F, et al (2004) Impact of FDG-PET on radiation therapy volume delineation in non-small-cell lung cancer. Int J Radiat Oncol Biol Phys. 59(1): 78–86. 10.1016/j.ijrobp.2003.10.044 15093902

[18] Wang S, Li J, Zang Y, Wang W, Li F, et al (2012) Measurement of Intra-Fraction Displacement of the Mediastinal Metastatic Lymph Nodes Using Four-Dimensional CT in Non-Small Cell Lung Cancer, Korean J Radiol. 13(4): 417–424. 10.3348/kjr.2012.13.4.417 PMC3384823 22778563

